# Randomized Evaluation of a Textile-Based Enhanced Radiation Protection Device Versus Control Shielding in the Cardiac Catheterization Laboratory

**DOI:** 10.1016/j.jscai.2026.105523

**Published:** 2026-07-14

**Authors:** Oskar Angerås, Pernilla I. Jonasson, Christian Dworeck, Gunilla Friberg, Dan Ioanes, Antros Louca, Jacob Odenstedt, Petur Petursson, Björn Redfors, Petronella Torild, Fanny Töpel, Maria E.V. Larsson, Truls Råmunddal

**Affiliations:** a Department of Molecular and Clinical Medicine, Institute of Medicine, University of Gothenburg, Gothenburg, Sweden; b Department of Thoracic Surgery and Cardiology, Sahlgrenska University Hospital, Gothenburg, Sweden; c Department of Medical Physics and Biomedical Engineering, Sahlgrenska University Hospital, Gothenburg, Sweden; d Department of Medical Radiation Sciences, Institute of Clinical Sciences, Sahlgrenska Academy at University of Gothenburg, Gothenburg, Sweden

**Keywords:** enhanced radiation protection device, occupational health, percutaneous coronary intervention, radiation protection

## Abstract

**Background:**

Occupational radiation exposure remains an inherent risk in cardiac catheterization laboratories. Conventional radiation protection relies on ceiling- and table-mounted shields combined with wearable lead-equivalent personal protective equipment, yet exposure persists and ergonomic burden remains substantial. Enhanced radiation protection devices (ERPD) aim to attenuate scatter radiation at the patient level, but randomized evaluations using comprehensive dosimetry are limited.

**Methods:**

In this prospective, single-center, cluster-randomized study, shielding strategies were alternated by week (6 ERPD vs 6 control weeks). A textile-based ERPD (Texray MasterPeace™) was compared with control shielding. Occupational radiation exposure to the first operator and scrub nurse was assessed using a 69-point thermoluminescent dosimetry array worn over personal protective equipment, capturing deep dose equivalent (Hp(10) for operator and scrub nurse roles. The primary end point was weekly cumulative Hp(10) normalized to patient radiation output. Secondary analyses included spatial dose distribution and accumulated Hp(10) over the study period.

**Results:**

A total of 190 procedures were included (ERPD n = 90; control n = 100). Baseline procedural characteristics, including dose area product and fluoroscopy time, were similar between groups. Mean weekly operator Hp(10) was 0.11 mSv with conventional shielding and 0.02 mSv with ERPD (mean difference −0.083 mSv; 95% CI −0.106 to −0.061; *P* < .001), corresponding to an 78% relative reduction. Scrub nurse exposure was similarly reduced (0.06 vs 0.01 mSv; 77% reduction). Findings were consistent with reductions across all anatomical regions.

**Conclusions:**

In a randomized real-world setting, MasterPeace ERPD substantially reduced occupational radiation exposure for both operators and scrub nurses, supporting its use as an adjunct to conventional shielding.

## Introduction

Occupational exposure to ionizing radiation remains an inherent and cumulative hazard for staff working in cardiac catheterization laboratories.^1^ Despite advances in fluoroscopy systems and procedural efficiency, cumulative exposure persists over time.^2^^,^^3^ The International Commission on Radiological Protection recommends an effective dose limit of 20 milliSievert (mSv) per year, averaged over 5 years, for occupational exposure, reinforcing the principle that radiation exposure should be kept as low as reasonably achievable).^4^^,^^5^ In occupational settings, personal dose equivalent Hp(10), at a depth of 10 mm, is used for monitoring purposes and as a conservative measure of effective dose.^5^

Conventional radiation protection relies primarily on wearable lead-equivalent personal protective equipment (PPE), supplemented by ceiling-suspended and table-mounted shields. While effective in attenuating scatter radiation, heavy aprons are associated with substantial musculoskeletal strain. Contemporary surveys indicate a high prevalence of chronic orthopedic symptoms among interventional cardiology staff, which could contribute to reduced procedural longevity and premature retirement.^6^ These competing occupational risks underscore the need for protection strategies that address both radiation safety and ergonomic health.^7^ Enhanced radiation protection devices (ERPDs) represent a paradigm shift by relocating the primary radiation barrier from the operator’s body to the procedural environment. MasterPeace ERPD (Texray AB) is a textile-based radiation protection system designed to attenuate scatter radiation at the patient level, while preserving procedural access.^8^, ^9^, ^10^ Early clinical evaluations of ERPDs have reported reductions in operator exposure of 60% to more than 90%.^11^, ^12^, ^13^ However, the majority of these studies are limited by nonrandomized designs, reliance on a few measurement points with real-time dosimeters, short evaluation periods, and incomplete anatomical dose mapping. Furthermore, detailed spatial dose distribution patterns, particularly across anatomically vulnerable regions, have not been evaluated using passive thermoluminescent dosimetry (TLD). To address these knowledge gaps, we conducted a randomized controlled trial comparing the ERPD with control shielding during routine coronary angiography and percutaneous coronary intervention (PCI). The primary objective was to compare occupational radiation exposure, measured as Hp(10) normalized to patient radiation output, between the ERPD and control shielding under randomized real-world procedural conditions, with dosimeters positioned outside the PPE. Secondary objectives included characterization of spatial radiation distribution patterns and estimation of annual occupational dose exposure.

## Materials and methods

### Study design

This is a prospective, single-center, cluster-randomized controlled study conducted over 12 consecutive weeks at Sahlgrenska University Hospital, Gothenburg, Sweden. The study evaluated occupational radiation exposure among catheterization laboratory staff during routine coronary angiography and PCI. The 12-week study period was divided into 6 predefined 2-week blocks. Within each block, 1 week was randomly assigned to ERPD (intervention) and the other to control shielding (control) using a computer-generated allocation sequence prepared prior to study initiation. Randomization occurred at the week level. All eligible procedures performed during each randomized week were analyzed according to the assigned shielding strategy. Dosimeters were worn by the primary operator and the scrub nurse. The primary operator was defined as the interventional cardiologist performing the procedure and positioned on the patient’s right side, closest to both the patient and the x-ray source. A total of 9 operators participated, sharing a single standardized overlay garment equipped with an array of TLDs, with dosimetry measurements obtained outside their PPE. The scrub nurse was positioned adjacent to the operator on the patient’s right side. In total, 16 nurses participated, also sharing a single standardized overlay garment equipped with an array of TLDs. Minor positional adjustments during procedures were permitted, reflecting routine clinical practice.

The study protocol was approved by the Swedish Ethical Review Authority (DNR 2024-04559-01). All participating staff members provided written informed consent. Because the study concerned occupational radiation protection and did not alter patient treatment, imaging protocols, or procedural strategy, the requirement for individual patient informed consent was waived by the ethics committee.

### Study population and procedural characteristics

All consecutive daytime diagnostic coronary angiography and PCI procedures performed during randomized weeks were eligible. Procedures performed outside regular working hours were excluded to ensure consistency in staffing and workflow. All procedures were performed in a single catheterization laboratory using a Philips Azurion angiography system (Philips Healthcare). The standard imaging protocol used fluoroscopy at 3.75 frames per second and cine acquisition at 7.5 frames per second. Imaging protocols and acquisition parameters remained identical throughout the study period.

Procedural strategy, vascular access site, imaging settings, and adjunctive device use were left to the discretion of the primary operator and reflected routine clinical practice. Nine interventional cardiologists and 16 scrub nurses participated during the study period and rotated between study arms according to the standard clinical schedule.

### Shielding strategies

#### Control: control shielding

During control weeks, control shielding was used, consisting of table-mounted shields, a right-sided patient-side scatter radiation shield, a ceiling-suspended lead-acrylic shield, and a reusable patient-side radiation-attenuating drape (Central Illustration).

**Central Illustration:**
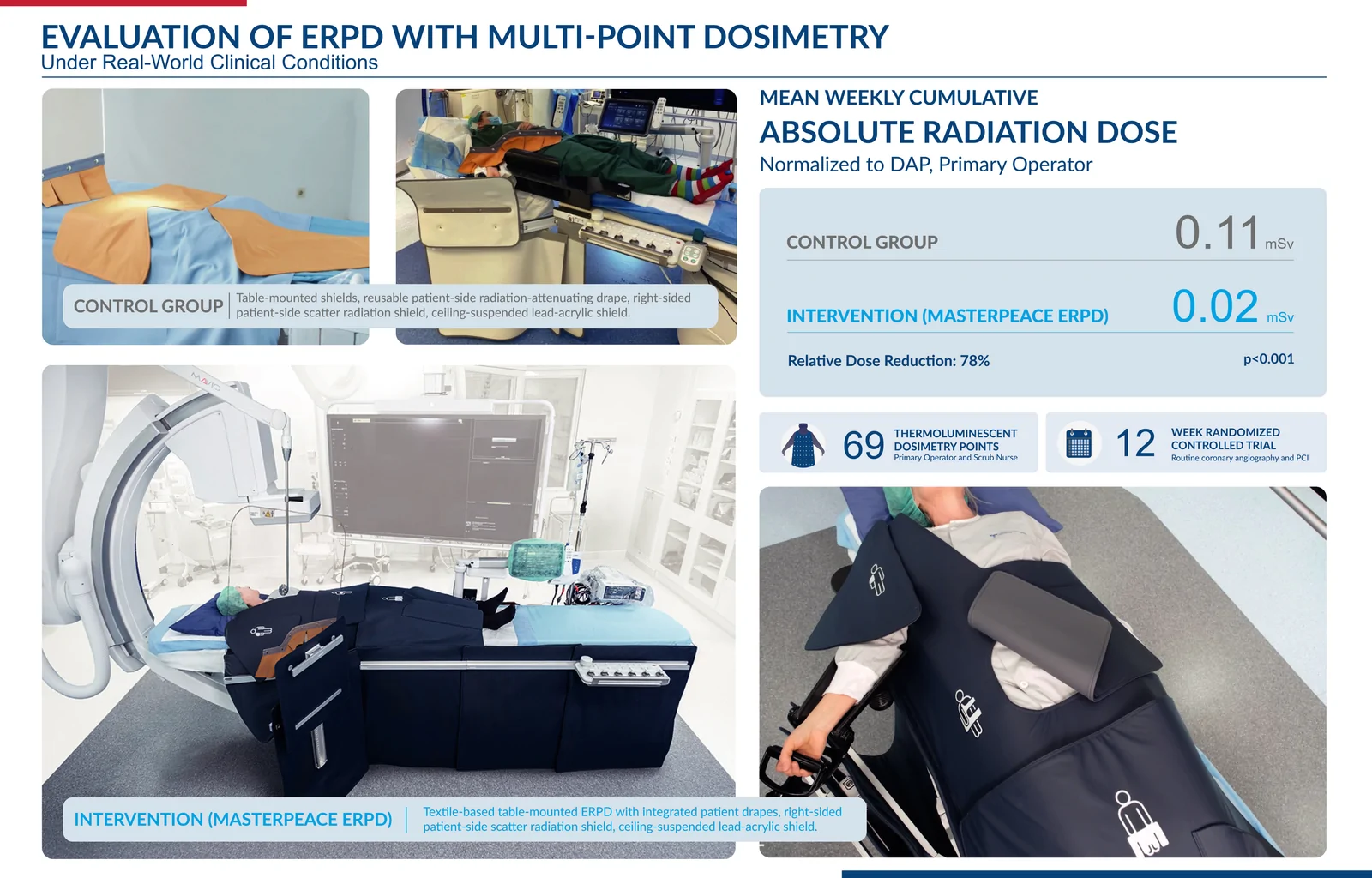
**Evaluation of ERPD with multi-point dosimetry.** Conventional shielding configuration (upper panels) and the textile-based MasterPeace ERPD configuration (lower panels) used in the study. The conventional shielding configuration consisted of table-mounted shields, a reusable patient-side radiation-attenuating drape, a right-sided patient-side scatter radiation shield, and a ceiling-suspended lead-acrylic shield. The MasterPeace ERPD configuration consisted of a textile-based table-mounted radiation protection system with integrated patient drapes used in combination with a right-sided patient-side scatter radiation shield and a ceiling-suspended lead-acrylic shield. ERPD, enhanced radiation protection device.

#### Intervention: MasterPeace ERPD

During intervention weeks, the table-mounted ERPD with integrated patient drapes was used in combination with the same right-sided patient-side scatter radiation shield and ceiling-suspended lead-acrylic shield.

All staff members wore PPE during both study arms to ensure safety redundancy. No other procedural workflows were modified.

Detailed device specifications are provided in the Supplemental Methods.

### Radiation dosimetry

Radiation exposure was assessed using passive TLDs, calibrated for Hp(10). To capture the cumulative weekly exposure per role, a single standardized overlay garment, worn over the PPE, equipped with the TLD array, was shared among all primary operators performing procedures during each randomized week; a corresponding garment was shared among the scrub nurses. The TLD arrays were worn upon entering and removed upon exiting the catheterization laboratory and were handled outside the procedure room. Due to the use of passive TLDs, which require post hoc readout, a consistent shielding strategy was applied within each measurement period.

The TLD array was worn externally, on top of the standard PPE, to enable exposure mapping of the radiation field reaching the operator. A total of 69 dosimeters were positioned on the primary operator and scrub nurse in a grid across the thorax, abdomen, pelvis, and lower extremities (Supplemental Figure S1). TLDs used are small and lightweight, and the multipoint array was designed to enable a more detailed spatial mapping of radiation exposure across anatomical regions than a single-point measurement. The reported radiation exposure reflects cumulative dose to the operator and scrub nurse roles, rather than individual-level measurements. Dosimeters were exchanged at the end of each week and analyzed. Background radiation was subtracted using control dosimeters stored outside the catheterization laboratory. Weekly cumulative radiation exposure was measured separately for operators and scrub nurses and corrected for the loss of backscatter from the PPE with a factor of 1.2. Technical details regarding dosimeter type, positioning, and processing are provided in the Supplementary Methods.

### Patient radiation metrics

For each procedure, the total dose area product (DAP, Gy·cm^2^) and fluoroscopy time were extracted directly from the angiography system. Weekly cumulative DAP was calculated to assess procedural comparability between study arms.

### Study end points

The primary end point was weekly cumulative scattered radiation exposure, measured as Hp(10) normalized to cumulative DAP, for the primary operator, representing the aggregated exposure recorded across all eligible procedures performed by primary operators during each study week, compared between shielding strategies using week as the unit of analysis (6 intervention weeks vs 6 control weeks). Weekly cumulative values were calculated from the mean of all the TLD positions (front and back) for operators and scrub nurses separately, analyzed after normalization to cumulative DAP.

Secondary end points included: (1) Weekly cumulative scattered radiation exposure normalized to cumulative DAP to the scrub nurse, representing the aggregated exposure recorded across all eligible procedures during each study week; (2) Accumulated scattered radiation exposure normalized to cumulative DAP during the study period to the primary operators and scrub nurses, respectively; (3) Anatomical distribution of radiation exposure normalized to cumulative DAP across the 69 measurement positions; and (4) Extrapolated annual radiation exposure normalized to cumulative DAP (mean weekly dose extrapolated to 800 procedures).

### Sample size considerations

The study was designed to detect a clinically meaningful reduction in scattered radiation exposure between shielding strategies. Based on prestudy measurements, weekly radiation levels were sufficient to provide reliable dosimeter readings above the detection level. Given the anticipated large effect size and the consistency of procedural volume, 6 randomized weeks per study arm were considered sufficient to detect meaningful differences in occupational dose.

### Statistical analysis

Continuous variables are presented as mean ± SD or median (IQR), as appropriate. Categorical variables are summarized as counts and percentages. For descriptive reporting, cumulative Hp(10) was calculated across the 6 intervention weeks and 6 control weeks. To account for differences in radiation exposure levels between study arms, Hp(10) was normalized to DAP by dividing weekly Hp(10) by weekly DAP and multiplying the result by the mean DAP across all 12 study weeks. Group comparisons were performed using Welch’s 2-sample *t* test to account for potential variance differences between arms. Mean weekly differences with 95% CI were reported. Six-week cumulative values and annualized radiation exposure (extrapolated to 800 procedures, assuming similar case mix) are presented descriptively and were not independently subjected to hypothesis testing. Statistical analyses were performed using two-sided tests, with *P* < 0.05 considered statistically significant.

## Results

### Procedural characteristics

A total of 190 procedures were performed during the 12-week study period: 90 during intervention weeks and 100 during control weeks. The distribution of procedure type (diagnostic coronary angiography vs PCI), chronic total occlusion interventions, and vascular access site was similar between study arms. Right radial access predominated in both groups. DAP and fluoroscopy time per procedure were similar between groups, indicating comparable procedural complexity. In 5 procedures, either the primary operator (4 cases) or the scrub nurse (1 case) did not use the dosimetry (Table 1).

**Table 1:** Procedural characteristics.

Variable	MasterPeace ERPD (n = 90^a^)	Control (n = 100^a^)	*P* value
Procedure type			.29^b^
Diagnostic coronary angiography only, %	36 (40%)	36 (36%)	
PCI, %	48 (53%)	60 (60%)	
CTO PCI, %	6 (7%)	4 (4%)	
Arterial access			.99^b^
Right radial artery, %	78 (87%)	86 (86%)	
Femoral artery, %	30 (33%)	34 (34%)	
DAP per procedure, Gy·cm^2^	9.98 (5.04-23.45)	11.31 (4.58-23.45)	.50^c^
Fluoroscopy time per procedure, min	10.79 (3.72-19.00)	9.24 (4.35-21.46)	.82^c^

Values are n (%) or median (IQR).

CTO, chronic total occlusion; DAP, dose area product; PCI, percutaneous coronary intervention.

a In 4 procedures, the primary operator was without dosimetry, in 1 procedure, the scrub nurse was without dosimetry.

b Chi-square test.

c Mann-Whitney *U* test.

### Primary end point: cumulative radiation exposure normalized to DAP (Table 2)

#### Primary operator

When analyzed using weeks as the unit of analysis (6 vs 6 weeks), the ERPD was associated with a statistically significant reduction in scatter radiation levels reaching the operatoŕs position. For operators, mean weekly Hp(10) was 0.11 mSv with control shielding and 0.02 mSv with ERPD, corresponding to a mean weekly difference of −0.083 mSv (95% CI, −0.106 to −0.061; *P* < .001), representing an 78% relative reduction.

**Table 2:** Study outcomes.

Outcome	Control shielding	MasterPeace + Ceiling Shield	Mean Difference (MasterPeace − Control)	95% CI	*P* value	Relative reduction
Primary end point (wk = unit; 6 vs 6)						
Weekly cumulative Hp(10), Operator (mSv/wk)	0.11	0.02	−0.083	−0.106 to −0.061	<.001	78%
Weekly cumulative Hp(10), Scrub nurse (mSv/wk)	0.06	0.01	−0.048	−0.071 to −0.026	.002	77%
Descriptive totals (no hypothesis test)						
Cumulative Hp(10), operator (mSv), 6 wk	0.63	0.14	—	—	—	78%
Cumulative Hp(10), scrub nurse (mSv), 6 wk	0.37	0.08	—	—	—	78%
Extrapolated annual exposure (800 procedures; descriptive)						
Annualized Hp(10), operator (mSv/y)	5.07	1.27	—	—	—	75%
Annualized Hp(10), scrub nurse (mSv/y)	2.94	0.68	—	—	—	77%

DAP, dose area product.

All values are normalized to DAP and are presented as means unless otherwise specified. Weekly comparisons were performed using Welch’s *t* test. DAP-standardized values reflect normalization to procedural radiation output (DAP). Six-week cumulative values are descriptive sums of weekly values. Annualized values assume 800 procedures with a similar case mix and radiation output.

The cumulative 6-week personal deep dose equivalent, Hp(10), for the primary operator was lower during ERPD weeks compared with control shielding weeks. Across the 6 intervention weeks, cumulative operator Hp(10) was 0.14 mSv, compared with 0.63 mSv across the 6 control weeks, corresponding to a relative reduction of 78%.

#### Scrub nurse

For the scrub nurse, mean weekly Hp(10) was 0.06 mSv with control shielding and 0.01 mSv with ERPD (mean weekly difference −0.048 mSv; 95% CI, −0.071 to −0.026; *P* = .002), corresponding to an 77% relative reduction. The cumulative 6-week occupational Hp(10) for the scrub nurse was 0.37 mSv with control shielding and 0.08 mSv with ERPD, corresponding to a relative reduction of 78% with the ERPD.

Crude Hp(10) measurements for the primary operator and scrub nurse, both as mean weekly exposure and cumulative exposure over the study period, showed similar patterns (Table S1).

### Spatial distribution of radiation dose (Figure 1)

Whole-body dosimetry using the 69-point TLD array demonstrated characteristic exposure patterns. Among operators, the highest exposure was observed over the left thoracoabdominal region, consistent with standard operator positioning relative to the x-ray source. Among scrub nurses, peak exposure was observed in the lower pelvic and thigh regions.

**Figure 1:**
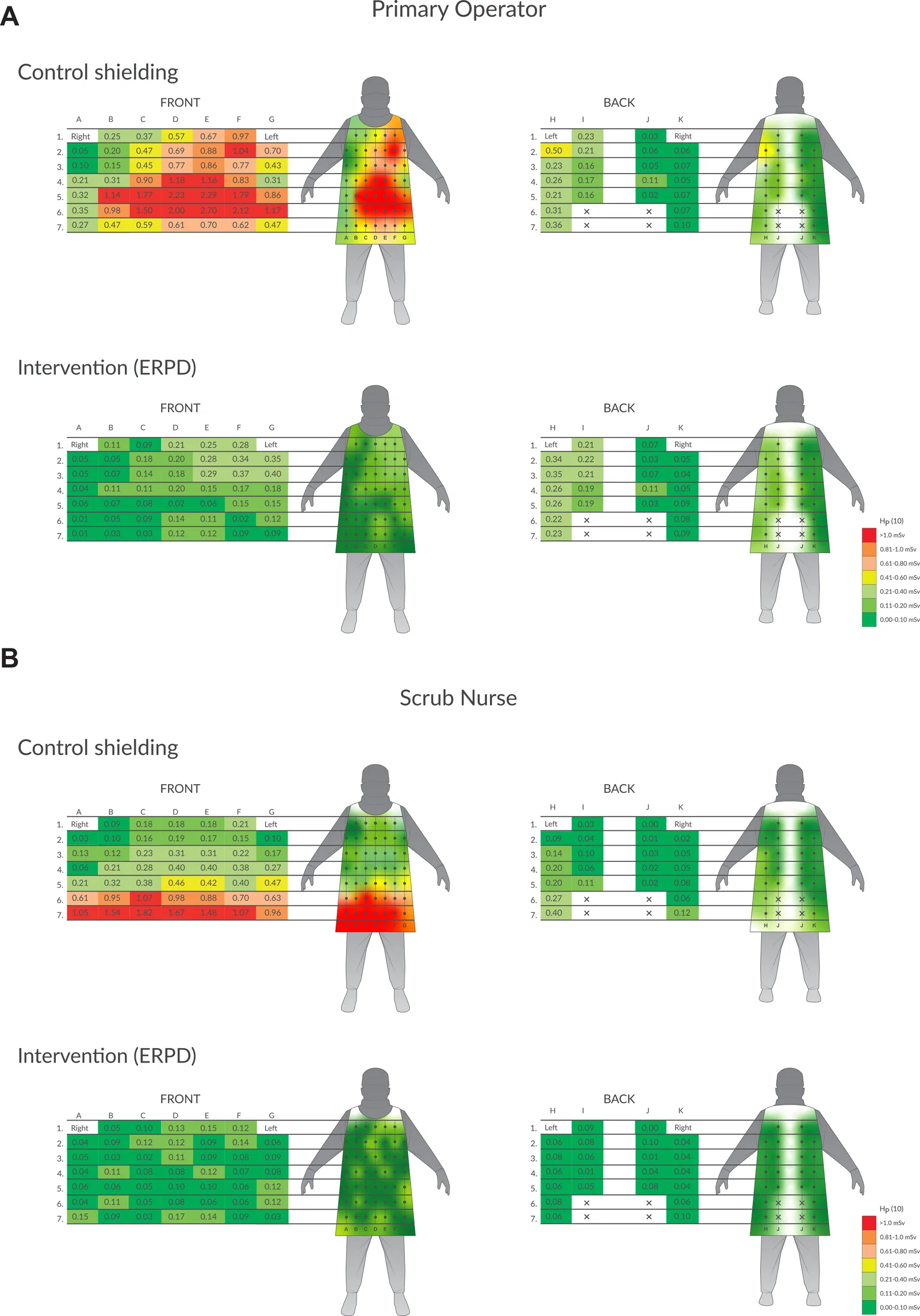
**Spatial distribution of scatter radiation exposure.** Illustrates the spatial distribution of the cumulative Hp(10) value using visual interpolation, derived from a TLD array with 69 measurement points on the outside of the personal protective equipment worn by the primary operator role (**A**) and the surgical nurse role (**B**) during procedures performed with respective Control shielding and the ERPD. The values represent the total radiation dose, normalized for DAP, at each measurement point over a 6-week period, aggregated across a total of 9 operators and sixteen surgical nurses who wore and shared the apron with the TLD array.

The ERPD was associated with reductions across all measured anatomical regions. No individual measurement point demonstrated increased exposure during intervention weeks compared with control weeks. For operators, the highest cumulative single-point dose recorded over the 6-week control period was 2.70 mSv at position 6E (corresponding to the left groin). During ERPD weeks, the highest corresponding cumulative value was 0.40 mSv at position 3G (left chest). For scrub nurses, the highest cumulative single-point dose during control weeks was 1.82 mSv at position 7C (right groin), compared with 0.17 mSv during ERPD weeks (position 7D, lower midline region).

#### Extrapolated annual exposure

To account for the difference in procedural volume during the study weeks (90 procedures in the ERPD group vs 100 in the control group), annual occupational exposure was extrapolated to a standardized volume of 800 procedures per year for both strategies. Based on mean weekly occupational exposure normalized for cumulative DAP, the extrapolated annual operator dose was 1.27 mSv/year during the ERPD use compared with 5.07 mSv/year with control shielding. Corresponding annual estimates for the scrub nurse were 0.68 mSv/year and 2.94 mSv/year, respectively (Table 2).

Analysis of localized peak radiation exposure using the standardized annual volume further highlighted the protective effect. For the primary operator, the highest single-point cumulative exposure (position 6E, left groin) corresponded to an annualized value of 21.60 mSv/year during control weeks, compared with 3.56 mSv/year at the highest point (position 3G, left chest) during ERPD weeks. For scrub nurses, the peak single-point exposure during control weeks corresponded to an annualized value of 14.56 mSv/year, compared with 1.51 mSv/year during ERPD weeks (Supplemental Figure S2).

## Discussion

In this cluster-randomized controlled study using TLD, MasterPeace ERPD was associated with significant reductions in occupational radiation exposure for both the primary operator and the scrub nurse compared with control shielding in a contemporary catherization laboratory environment. The magnitude of dose reduction was consistent across anatomical regions and remained evident after normalization to procedural radiation output (DAP). To our knowledge, this represents one of the first randomized evaluations of an ERPD incorporating multipoint passive dosimetry under real-world clinical conditions.

Conventional radiation protection in the catheterization laboratory relies predominantly on wearable lead-equivalent PPE, supplemented by ceiling-suspended and table-mounted shields. Although effective in attenuating scatter radiation, heavy aprons are associated with substantial musculoskeletal strain. Survey data demonstrate a high prevalence of chronic neck, back, and joint disorders among interventional cardiology staff, underscoring the ergonomic burden of long-term lead use.^6^^,^^14^^,^^15^ Enhanced radiation protection devices, including MasterPeace ERPD, aim to relocate the primary radiation barrier from the operator’s body to the procedural environment. By attenuating scatter radiation at its source, such systems may reduce occupational exposure while preserving procedural access.^4^

In the present study, the ERPD was associated with a marked reduction in scatter radiation reaching the operator, achieving levels significantly lower than those measured during control weeks. All measurements in both study arms were performed external to the wearable PPE, and this investigation was not designed to evaluate the reduction or complete elimination of PPE. Although the observed magnitude of dose reduction supports the feasibility of substantially decreasing reliance on PPE, the safety, practicality, and long-term implications of apron-free practice require dedicated prospective evaluation, balancing adherence to the ALARA principle against the growing recognition of occupational musculoskeletal injury associated with prolonged use of heavy protective garments.^16^

Several other ERPDs have been evaluated in recent years, including Rampart (Rampart IC), EggNest (Egg Medical Inc), and Protego (Protego® Radiation Protection System; Image Diagnostics, Inc). Published reports have described substantial reductions in operator radiation exposure, frequently ranging from approximately 60% to more than 90%.^11^, ^12^, ^13^^,^^17^, ^18^, ^19^ However, important methodological differences limit direct quantitative comparison. Many prior evaluations have relied on real-time dosimetry with few measurement points, nonrandomized study designs, and short observation periods. In contrast, the present study incorporated cluster randomization and a 69-point passive TLD array, enabling comprehensive exposure mapping over multiple randomized weeks. This approach reduces susceptibility to measurement bias and provides a more robust assessment of radiation exposure.

Beyond shielding performance, ERPD platforms differ in structural design and workflow integration. Some systems require floor-mounted cabins or rigid enclosures positioned around the procedural table, which may increase setup complexity and limit flexibility during procedures. In high-volume or emergent settings, such factors may influence real-world adoption. The MasterPeace ERPD was designed for rapid deployment while preserving standard operator positioning and procedural access.^9^ The table-mounted shield remains in place throughout, and only patient drapes are applied for each procedure as part of routine workflow, without the need for additional personnel or apparent impact on procedural turnover. In situations requiring immediate patient access, the patient drapes can be rapidly removed without additional personnel, and no interference with urgent clinical management is observed. However, the impact of removing the patient drapes on personnel radiation exposure has not been evaluated in this study. Formal comparisons of setup time, workflow efficiency, and ergonomic impact between ERPD platforms have not been performed, and head-to-head comparisons should therefore be interpreted cautiously given differences in laboratory configuration, imaging technology, procedural complexity, and dosimetry methodology.

The use of 69 passive TLDs enabled a more detailed characterization of spatial exposure patterns from the upper part of the chest to above the knee. As expected, operators demonstrated left-sided predominance of exposure, consistent with standard laboratory geometry and previously reported asymmetric distribution of scattered radiation. Scrub nurses exhibited relatively higher exposure in lower pelvic and thigh regions, reflecting procedural positioning.

Importantly, in this study, when using the ERPD, none of the 69 measurement positions were associated with an increased dose exposure compared with control shielding. Furthermore, extrapolated annual radiation dose levels derived from each measurement position remained well below established occupational radiation exposure limits. The uniform attenuation observed across all anatomical regions, without compensatory increases at any site, suggests that effective environmental shielding can substantially reduce reliance on wearable lead protection. Although this study was not designed to evaluate the complete elimination of PPE, the absence of localized high-dose regions supports further investigation into structured strategies involving reduced apron weight or selective apron-free practice under controlled conditions and appropriate dosimetry monitoring. This expanded radiation exposure mapping approach provides a more granular assessment of radiation exposure than single-dosimeter measurements and may better approximate cumulative biological risk^20^^,^^21^ and, in our opinion, should be used, as in future evaluations and comparisons of ERPDs.^22^

By substantially lowering occupational radiation exposure while maintaining procedural access, MasterPeace ERPD may represent an important step toward improving occupational safety in the contemporary catheterization laboratory. Although the present study was not designed to evaluate clinical outcomes, sustained reductions in occupational radiation exposure may be expected to reduce cumulative radiation-related health risks over time. In addition to radiation attenuation, the potential ergonomic benefits associated with reduced reliance on heavy PPE and their impact on long-term musculoskeletal health warrant further prospective investigation.

### Limitations

This was a single-center study conducted in a modern catheterization laboratory with relatively low baseline radiation levels, which may limit generalizability. Randomization occurred at the week level, and the total number of randomized clusters was limited. Although sensitivity analyses supported the primary findings, residual confounding cannot be entirely excluded. Radiation doses to the head, lens, and thyroid gland were not included in this study. However, the ceiling-suspended lead-acrylic shield, used in both study groups, has been shown to offer protection of these areas.^23^^,^^24^ The dosimetry system was not designed to provide organ-specific dose estimates, as measurements were performed external to PPE and not calibrated for individual organ assessment. The study did not include direct head-to-head comparisons with other ERPD platforms, nor did it assess musculoskeletal outcomes or the safety of apron-free implementation strategies. Annual dose estimates were extrapolated from weekly data and assume stable procedural volume and case mix.

## Conclusion

In this cluster-randomized study using multipoint TLD measurements, MasterPeace ERPD was associated with significant reductions in occupational radiation exposure for both the primary operator and the scrub nurse compared with control shielding. These findings support the idea of ERPDs as an important strategy to improve occupational ergonomics in interventional cardiology, to a radiation dose level comparable to conventional shielding, including wearable PPE.

By attenuating scatter radiation at the source, this shielding strategy offers a potential pathway to improve the occupational environment in the catheterization laboratory. These results provide a foundation for future evaluations of structured strategies to reduce the musculoskeletal burden of heavy wearable PPE while maintaining high standards of radiation safety.

## CRediT authorship contribution statement

**Oskar Angerås:** Writing – review & editing, Writing – original draft, Visualization, Project administration, Methodology, Investigation, Formal analysis, Conceptualization. **Pernilla I. Jonasson:** Writing – review & editing, Validation, Methodology, Investigation, Data curation. **Christian Dworeck:** Writing – review & editing, Investigation. **Gunilla Friberg:** Writing – review & editing, Investigation. **Dan Ioanes:** Writing – review & editing, Investigation. **Antros Louca:** Writing – review & editing, Investigation. **Jacob Odenstedt:** Writing – review & editing, Investigation. **Petur Petursson:** Writing – review & editing, Investigation. **Björn Redfors:** Writing – review & editing, Software, Methodology, Data curation. **Petronella Torild:** Writing – review & editing, Project administration, Investigation. **Fanny Töpel:** Writing – review & editing, Investigation. **Maria E.V. Larsson:** Writing – review & editing, Validation, Project administration, Methodology, Investigation, Data curation, Conceptualization. **Truls Råmunddal:** Writing – review & editing, Visualization, Resources, Project administration, Methodology, Investigation, Funding acquisition, Conceptualization.

## Declaration of competing interest

Oskar Angerås and Truls Råmunddal are co-inventors on a patent related to the MasterPeace ERPD and are shareholders in Texray AB. All other authors reported no financial interests.

## Funding sources

The study has been funded by Medtech4Health/Vinnova, Sweden.

## Declaration of Generative AI and AI-Assisted Technologies in the Writing Process

During the preparation of this work, the authors used ChatGPT (OpenAI) in order to assist with language editing and refinement of the manuscript text. After using this tool, the authors reviewed and edited all content and take full responsibility for the content of the publication.
