# Supplementary material for: Randomized Evaluation of a Textile-Based Enhanced Radiation Protection Device Versus Control Shielding in the Cardiac Catheterization Laboratory
Source: J Soc Cardiovasc Angiogr Interv. 2026 Jul 14;5(9):105523. doi: 10.1016/j.jscai.2026.105523 (PMC13591509; doi:10.1016/j.jscai.2026.105523)

”Yellow apron”

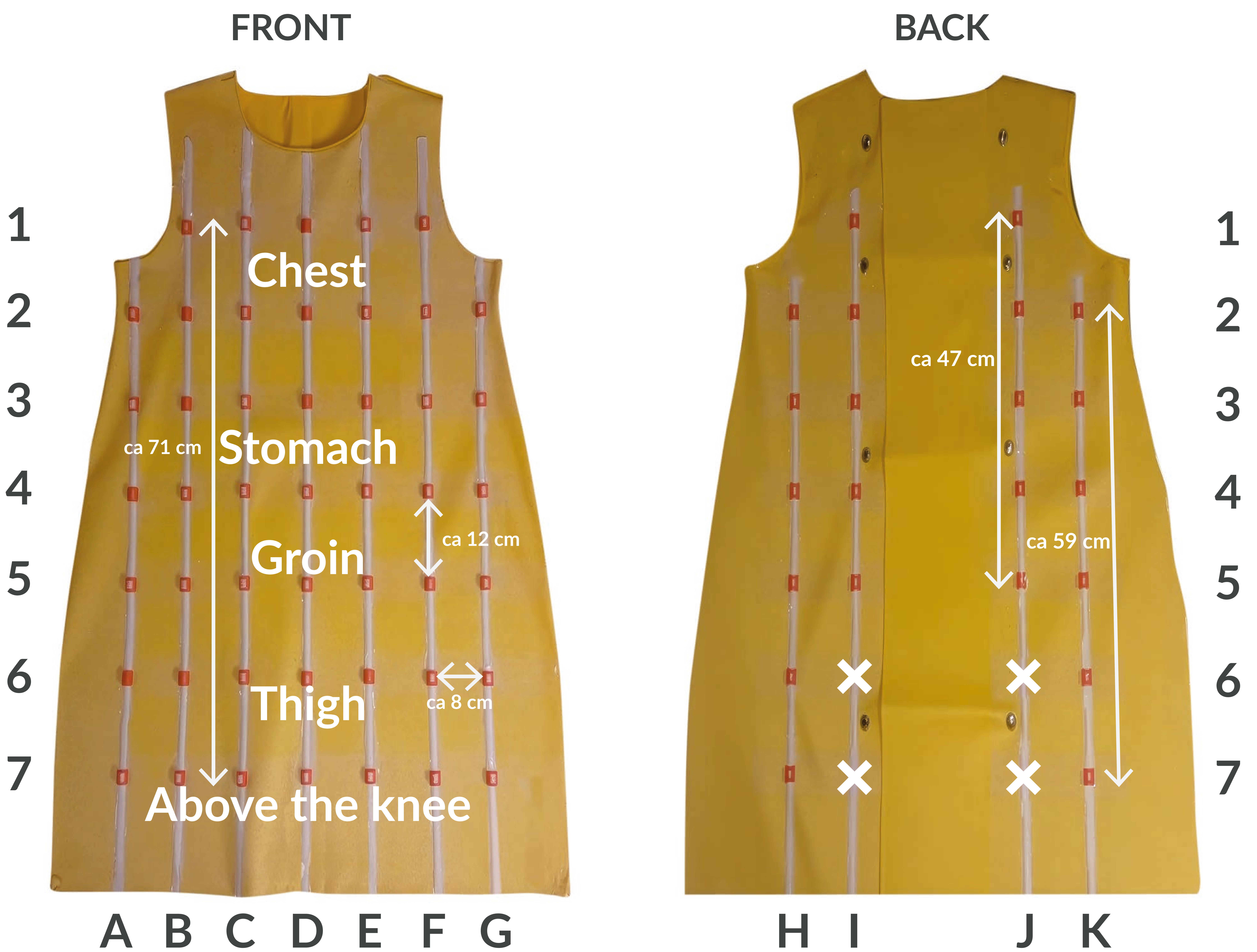

Supplement: Supplementary file 1 [file mmc2.pdf]
