# Supplementary material for: Randomized Evaluation of a Textile-Based Enhanced Radiation Protection Device Versus Control Shielding in the Cardiac Catheterization Laboratory
Source: J Soc Cardiovasc Angiogr Interv. 2026 Jul 14;5(9):105523. doi: 10.1016/j.jscai.2026.105523 (PMC13591509; doi:10.1016/j.jscai.2026.105523)
Supplement: Supplementary file 2 [file mmc3.pdf]

(A) Primary Operator

Control shielding

FRONT

	A	B	C	D	E	F	G
1.	Right	2.00	2.96	4.56	5.36	7.76	Left
2.	0.40	1.60	3.76	5.52	7.04	8.32	5.60
3.	0.80	1.20	3.60	6.16	6.88	6.16	3.44
4.	1.68	2.48	7.20	9.44	9.28	6.64	2.48
5.	2.56	9.12	14.16	17.84	18.32	14.32	6.88
6.	2.80	7.84	12.00	16.00	21.60	16.96	9.36
7.	2.16	3.76	4.72	4.88	5.60	4.96	3.76

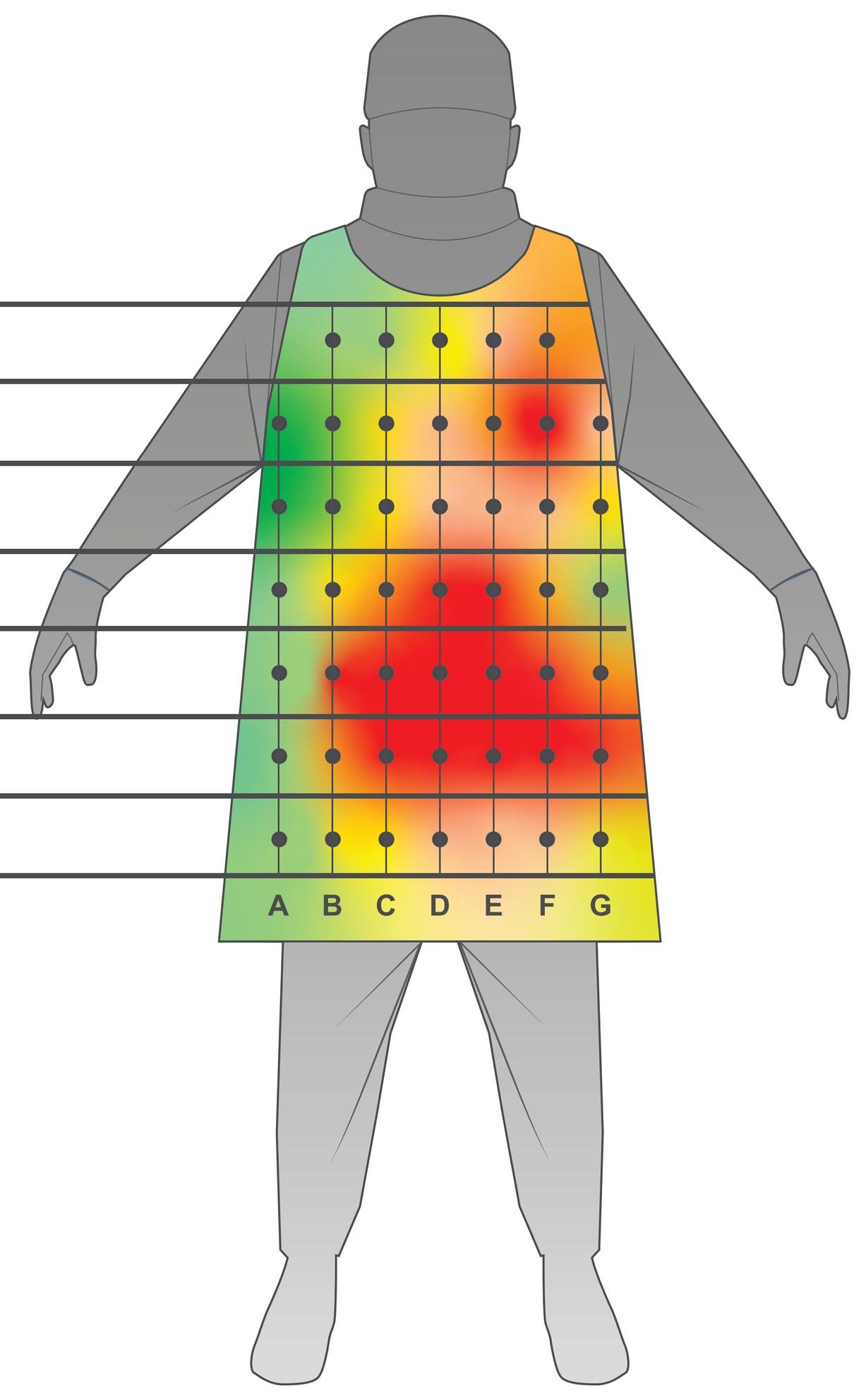

BACK

	H	I	J	K
1.	Left	1.84	0.24	Right
2.	4.00	1.68	0.48	0.48
3.	1.84	1.28	0.40	0.56
4.	2.08	1.36	0.88	0.40
5.	1.68	1.28	0.16	0.56
6.	2.48	×	×	0.56
7.	2.88	×	×	0.80

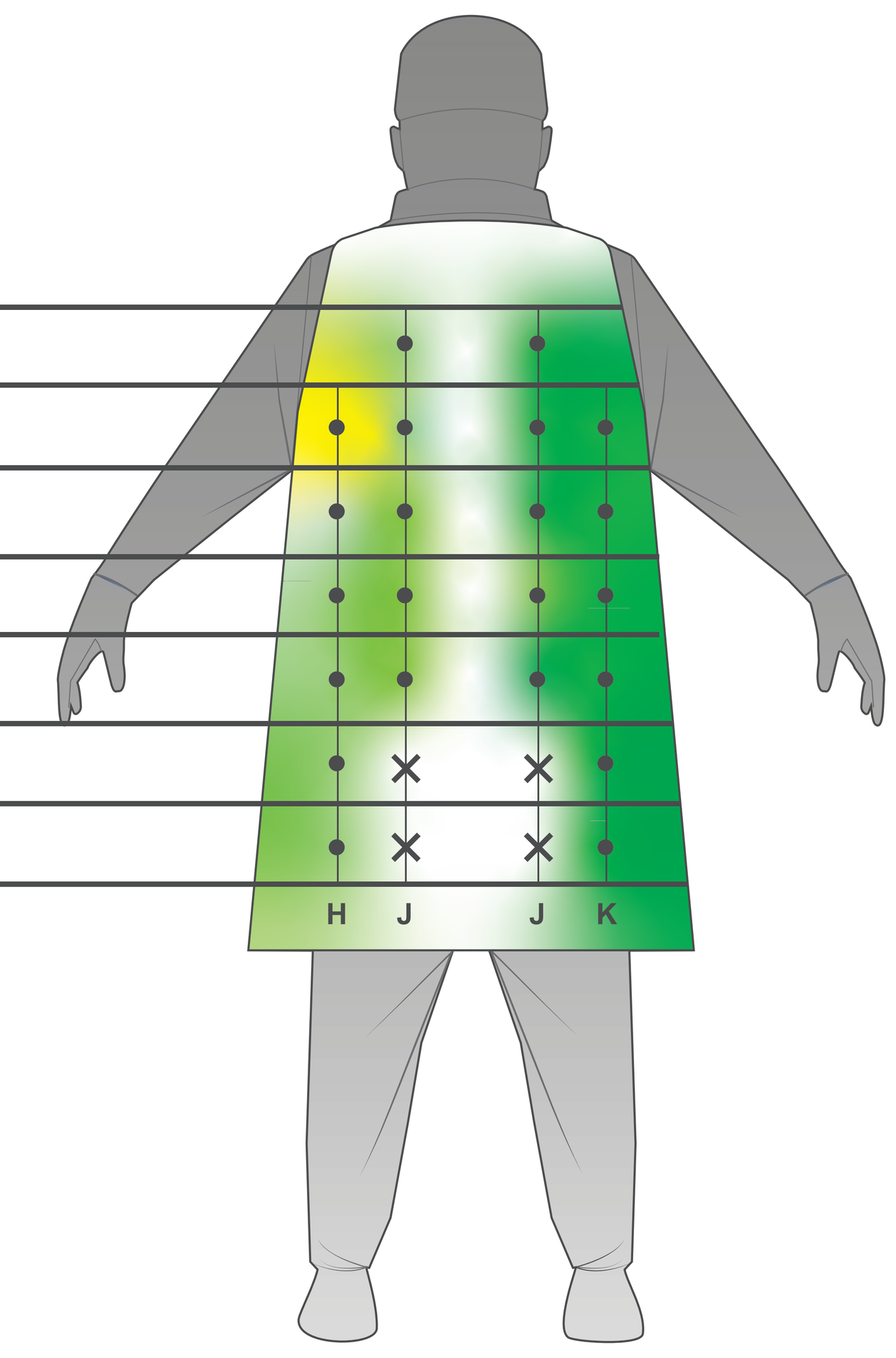

Intervention (ERPD)

FRONT

	A	B	C	D	E	F	G
1.	Right	0.98	0.80	1.87	2.22	2.49	Left
2.	0.44	0.44	1.60	1.78	2.49	3.02	3.11
3.	0.44	0.62	1.24	1.60	2.58	3.29	3.56
4.	0.36	0.98	0.98	1.78	1.33	1.51	1.60
5.	0.53	0.62	0.71	0.18	0.53	1.33	1.33
6.	0.09	0.44	0.80	1.24	0.98	0.18	1.07
7.	0.09	0.27	0.27	1.07	1.07	0.80	0.80

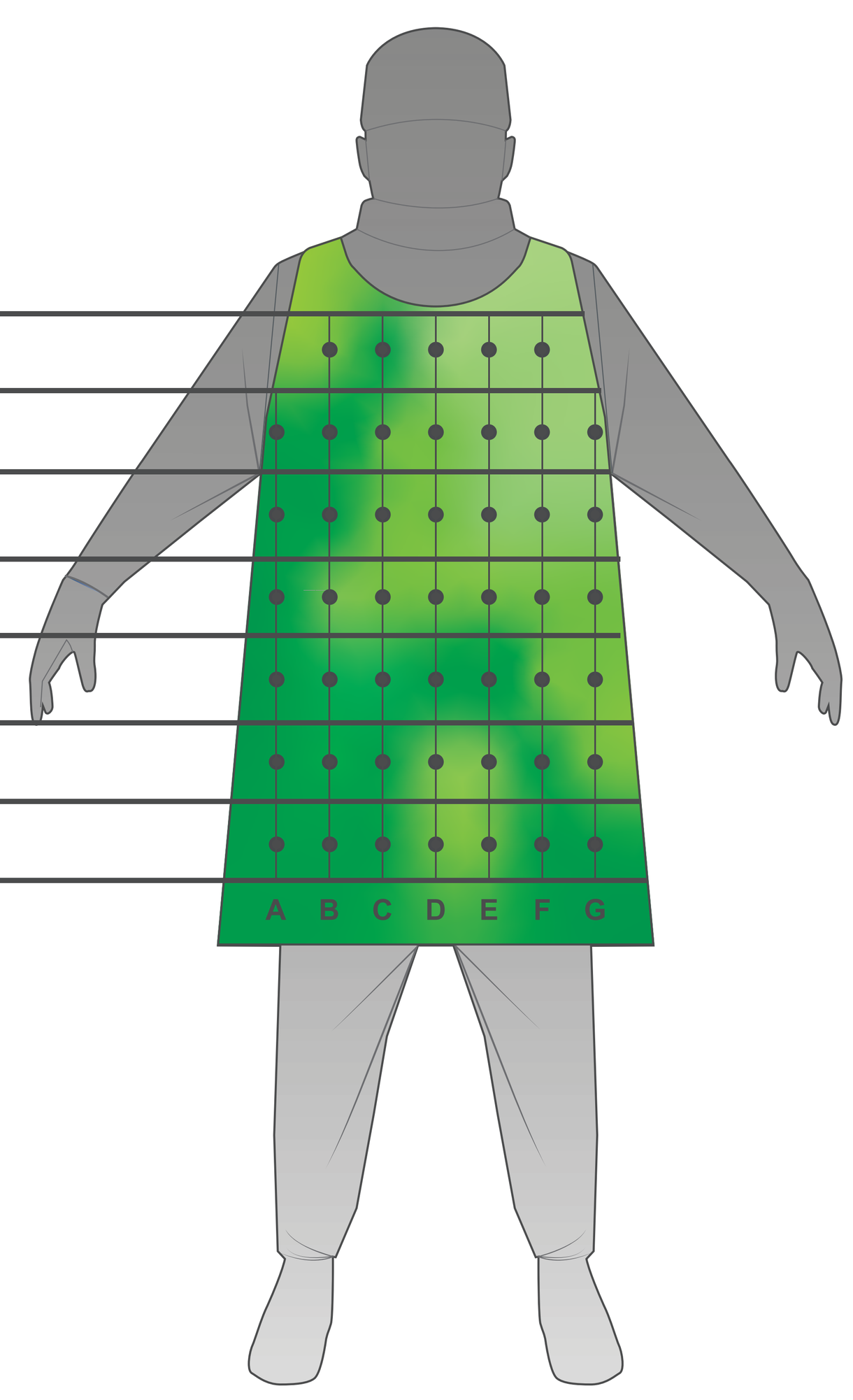

BACK

	H	I	J	K
1.	Left	1.87	0.62	Right
2.	3.02	1.96	0.27	0.44
3.	3.11	1.87	0.62	0.36
4.	2.31	1.69	0.98	0.44
5.	2.31	1.69	0.27	0.80
6.	1.96	×	×	0.71
7.	2.04	×	×	0.80

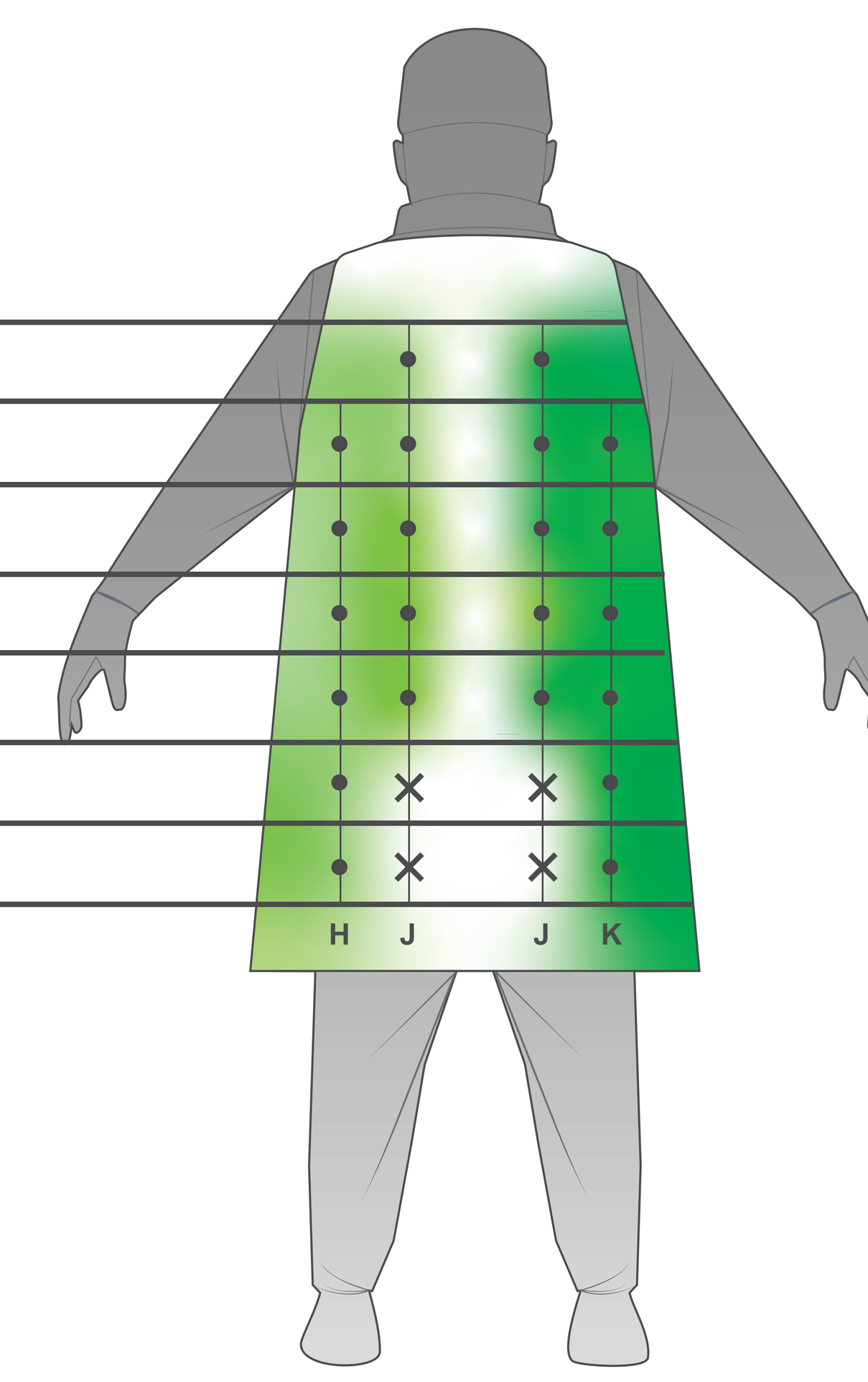

Hp (10)
- >8.00 mSv
- 6.17-7.99 mSv
- 4.73-6.16 mSv
- 3.57-4.72 mSv
- 1.61-3.56 mSv
- 0.96-1.78 mSv
- 0.00-0.95 mSv

(B) Scrub Nurse

Control shielding

FRONT

	A	B	C	D	E	F	G
1.	Right	0.72	1.44	1.44	1.44	1.68	Left
2.	0.24	0.80	1.28	1.52	1.36	1.20	0.80
3.	1.04	0.96	1.84	2.48	2.48	1.76	1.36
4.	0.48	1.68	2.24	3.20	3.20	3.04	2.16
5.	1.68	2.56	3.04	3.68	3.36	3.20	3.76
6.	4.88	7.60	8.56	7.84	7.04	5.60	5.04
7.	8.40	12.32	14.56	13.36	11.84	8.56	7.68

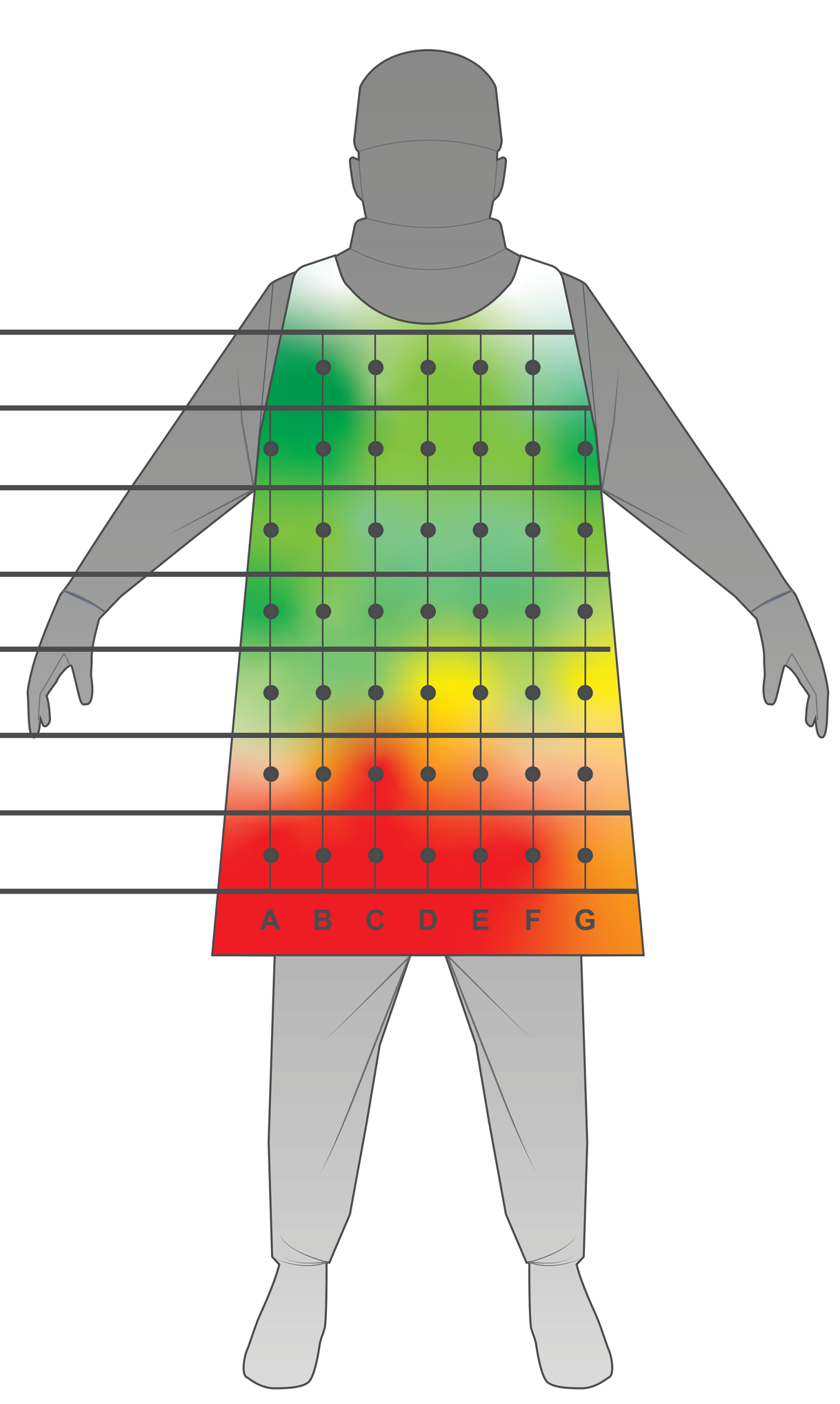

BACK

	H	I	J	K
1.	Left	0.24	0.00	Right
2.	0.72	0.32	0.08	0.16
3.	1.12	0.80	0.24	0.40
4.	1.60	0.48	0.16	0.40
5.	1.60	0.88	0.16	0.64
6.	2.16	×	×	0.48
7.	3.20	×	×	0.96

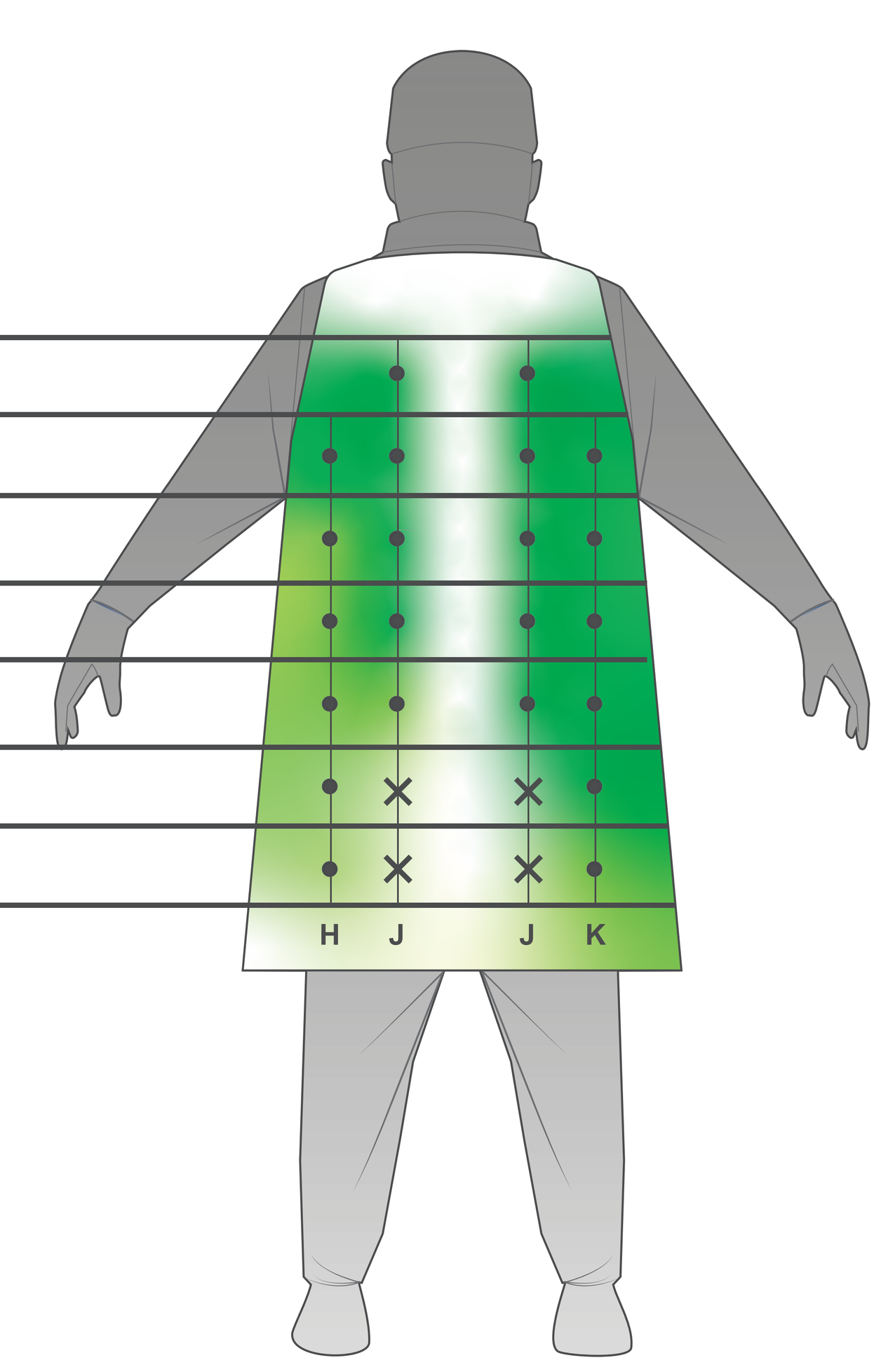

Intervention (ERPD)

FRONT

	A	B	C	D	E	F	G
1.	Right	0.44	0.89	1.16	1.33	1.07	Left
2.	0.36	0.80	1.07	1.07	0.80	1.24	0.53
3.	0.44	0.27	0.18	0.98	0.80	0.71	0.80
4.	0.36	0.98	0.71	0.71	1.07	0.62	0.71
5.	0.53	0.53	0.44	0.89	0.89	0.53	1.07
6.	0.36	0.98	0.44	0.71	0.53	0.53	1.07
7.	1.33	0.80	0.27	1.51	1.24	0.80	0.27

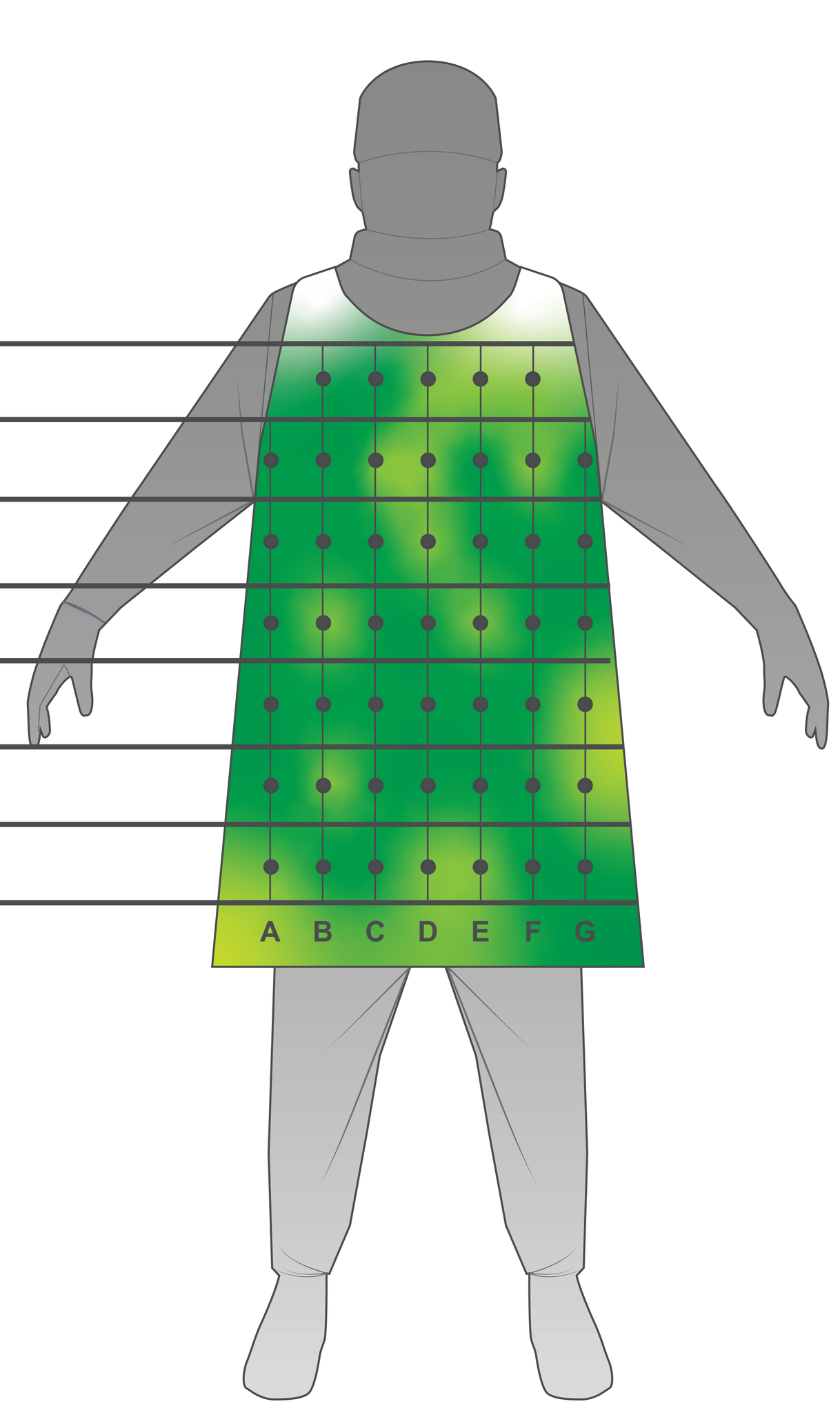

BACK

	H	I	J	K
1.	Left	0.80	0.00	Right
2.	0.53	0.71	0.89	0.36
3.	0.71	0.53	0.09	0.36
4.	0.53	0.09	0.36	0.36
5.	0.53	0.44	0.71	0.36
6.	0.71	×	×	0.53
7.	0.53	×	×	0.89

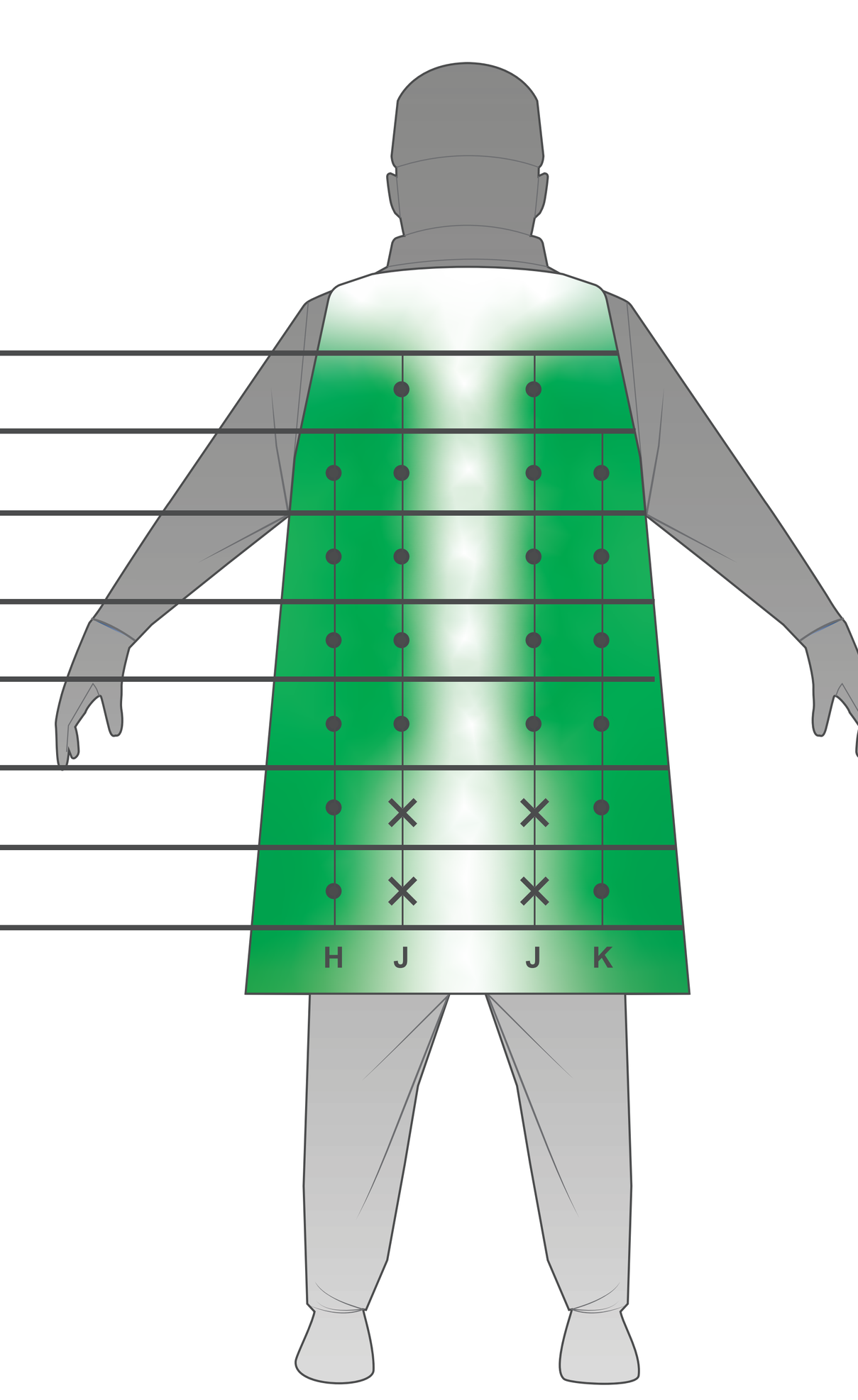

Hp (10)
- >8.00 mSv
- 6.17-7.99 mSv
- 4.88-6.16 mSv
- 3.57-4.79 mSv
- 1.79-3.56 mSv
- 0.96-1.78 mSv
- 0.00-0.95 mSv
