# Supplementary material for: Randomized Evaluation of a Textile-Based Enhanced Radiation Protection Device Versus Control Shielding in the Cardiac Catheterization Laboratory
Source: J Soc Cardiovasc Angiogr Interv. 2026 Jul 14;5(9):105523. doi: 10.1016/j.jscai.2026.105523 (PMC13591509; doi:10.1016/j.jscai.2026.105523)
Supplement: Supplementary file 3 [file mmc1.docx]

Supplementary Appendix

# Device Specifications

Control (Central illustration):

- 92 cm wide table shield (312/E-013, Kenex Ltd, Essex, England) (0.5 mm Pb equivalent)
- Scatter radiation shield (STARTable, Adept Medical Ltd, Auckland, New Zeeland) (0.5 mm Pb equivalent)
- Ceiling-suspended lead-acrylic shield (OT54001, MAVIG GmbH, Munich, Germany) (0.5 mm Pb equivalent)
- Reusable patient-side drape (ST-FZ/ST-RZ series, MAVIG GmbH, Munich, Germany) (0.5 mm Pb equivalent)

Intervention (Central illustration):

- Textile-based table-mounted with integrated patient drapes structural shielding system (MasterPeace ERPD, Texray AB, Gothenburg, Sweden) (0.25-0.5 mm Pb equivalent)
- Scatter radiation shield (STARTable, Adept Medical Ltd, Auckland, New Zealand) (0.5 mm Pb equivalent)
- Ceiling-suspended lead-acrylic shield (OT54001, MAVIG GmbH, Munich, Germany) (0.5 mm Pb equivalent)

Lead-equivalent values are manufacturer specifications.

# Dosimetry Configuration

Occupational radiation exposure was assessed using **69 passive thermoluminescent dosimeters (LiF:Mg,Ti)** (DXT-RAD, TLD-100, Wristband holder, Cap (42 mg/cm²), Thermo Fisher Scientific, Waltham, MA, USA). Dosimeters were positioned at predefined anatomical locations on a dedicated overlay garment worn **outside the lead-equivalent protective apron** of both the primary operator and the scrub nurse (Figure S3).

The dosimeter array covered anterior and posterior anatomical regions including the thorax, abdomen, pelvis, and lower extremities (Figure S3). All measurements were reported as **personal dose equivalent Hp(10)** in millisieverts (mSv).

Dosimeters were calibrated for radiation quality N80 according to ISO 4037-1:2021 and ISO 4037-3:2021. Measurement uncertainty arises from both calibration and measurement conditions in the clinical setting.

Supplementary Figures

Figure S1. Dosimetry overlay garment with 69 TLD positions.

Figure S2. Spatial distribution of scatter radiation exposure extrapolated to assumed annual volume of 800 procedures.

**Figure S1. Layout of the 69-point thermoluminescent dosimetry (TLD) array on the overlay garment.**
Illustration of dosimeter placement on the dedicated overlay garment used for radiation measurements. Measurement positions were organized in a grid (A–G anteriorly and H–K posteriorly; rows 1–7), enabling spatial mapping of radiation exposure so that anatomical locations on the anterior (front) and posterior (back) surfaces, covered the chest, abdomen, groin, thighs, and regions above the knee. Distances between measurement points and anatomical coverage are indicated. Positions marked with “X” on the posterior side were not available for measurement due to garment design constraints.

Figure S2. Spatial distribution of scatter radiation exposure.
A visual interpolation based on measurement data, illustrating the spatial distribution of cumulative Hp(10) extrapolated to assumed annual procedural volume of 800 procedures measured using a 69-point TLD array positioned outside the personal protective equipment of the first operator (A) and scrub nurse (B) during the Control shielding and the ERPD, respectively.

Table S1. Crude dose measurements

| **Outcome** | **Control Shielding** | **MasterPeace + Ceiling Shield** | **Mean Difference (Med − Conv)** | **95% CI** | **p-value** | **Relative Reduction** |
| --- | --- | --- | --- | --- | --- | --- |
| **Crude accumulated Hp(10) (week = unit; 6 vs 6)** |  |  |  |  |  |  |
| Weekly cumulative Hp(10), **Operator**(mSv/week) | 0.11 | 0.02 | −0.095 | −0.143 to −0.047 | 0.003 | 84% |
| Weekly cumulative Hp(10), **Scrub nurse** (mSv/week) | 0.07 | 0.01 | −0.057 | −0.088 to −0.026 | 0.004 | 85% |
| **Descriptive totals (no hypothesis test)** |  |  |  |  |  |  |
| Cumulative Hp(10), **Operator** (mSv), 6 weeks | 0.68 | 0.11 | — | — | — | 84% |
| Cumulative Hp(10), **Scrub nurse**(mSv), 6 weeks | 0.39 | 0.06 | — | — | — | 85% |
| **Extrapolated annual exposure (800 procedures; descriptive)** |  |  |  |  |  |  |
| Annualized Hp(10), **Operator**(mSv/year) | 5.44 | 0.98 | — | — | — | 82% |
| Annualized Hp(10), **Scrub nurse**(mSv/year) | 3.12 | 0.53 | — | — | — | 83% |
| Annualized Hp(10), **DAP-standardized Scrub nurse**(mSv/year) | 2.96 | 0.72 | — | — | — | 76% |
